# An Unusual Presentation of Cholecystoduodenal Fistula: Abdominal Pain out of Proportion to Exam

**DOI:** 10.5811/cpcem.2019.4.42686

**Published:** 2019-05-29

**Authors:** Ryan McCreery, Matthew Meigh

**Affiliations:** Orange Regional Medical Center, Department of Emergency Medicine, Middletown, New York

## Abstract

Cholecystoduodenal fistula (CDF) is a rare complication of gallbladder disease. Clinical presentation is variable, and preoperative diagnosis is challenging due to the non-specific symptoms of CDF. We discuss a 61-year-old male with a history of atrial fibrillation who presented with severe abdominal pain out of proportion to exam. The patient was diagnosed promptly and successfully managed non-operatively. This case presentation emphasizes the need to maintain a broad differential diagnosis for abdominal pain out of proportion to exam, with the possibility of a biliary-enteric fistula as a possible cause. It also stresses the importance of a multimodality imaging approach to arrive at a final diagnosis.

## CASE PRESENTATION

**Image 1:**
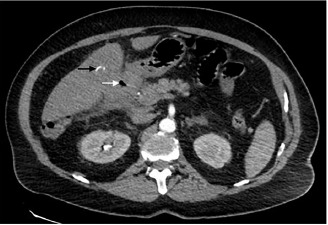
Computed tomography angiography (axial view) demonstrating a wide soft tissue tract between the gallbladder and proximal duodenum (white arrow). Calcified gallstones seen entering the proximal duodenum and along the undersurface of the right hepatic lobe (black arrow).

**Image 2:**
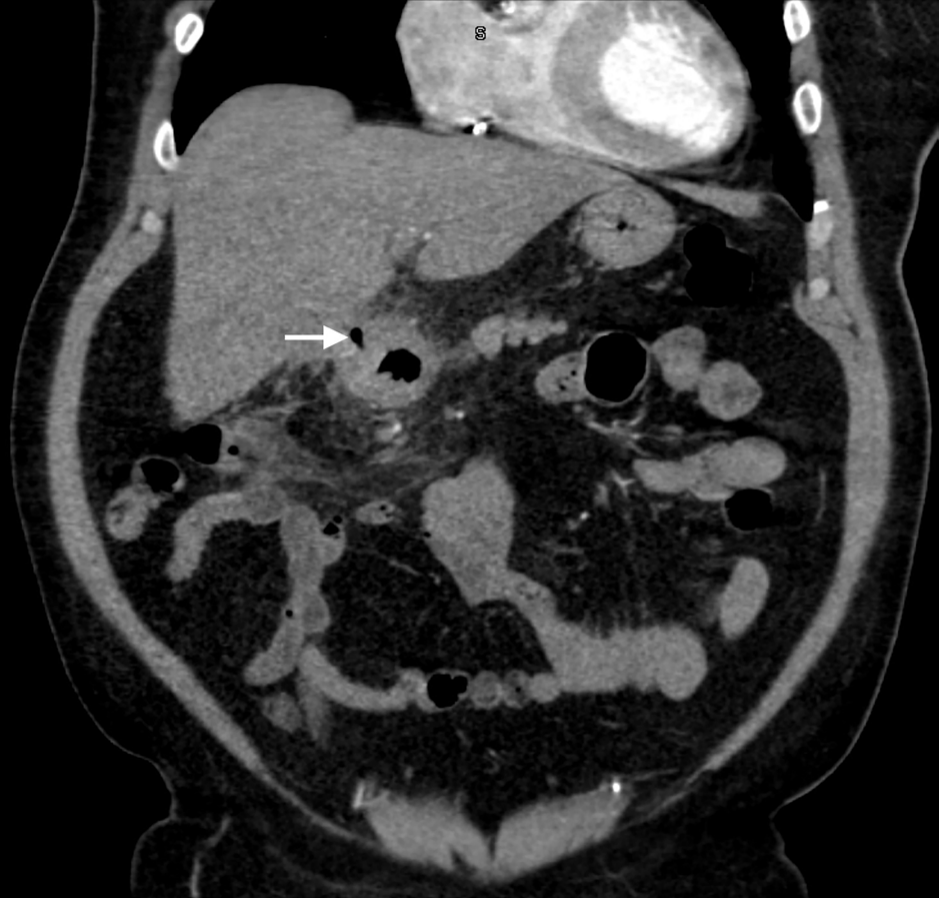
Computed tomography angiography (coronal view) demonstrating a wide, soft tissue tract between the gallbladder and proximal duodenum, containing a small amount of air (white arrow).

**Image 3:**
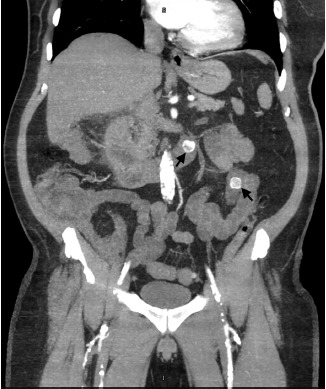
Computed tomography angiography (coronal view) demonstrating two rim-calcified gallstones within the proximal jejunum (black arrows).

A 61-year-old male with a history of atrial fibrillation presented to our emergency department with intermittent, post-prandial abdominal pain over the prior week. The patient localized the pain to the epigastric region and reported associated diarrhea. On examination, he was afebrile but appeared markedly distressed with an irregularly irregular rhythm at 108 beats per minute. Abdominal exam revealed mild generalized tenderness without guarding or rebound. Laboratory study results were unremarkable. Right upper quadrant ultrasound revealed gallstones with gallbladder wall thickening measuring up to 9.0 millimeters. In conjunction with the ultrasound findings, computed tomography (CT) angiography of the abdomen and pelvis revealed acute cholecystitis with gallbladder adherence to the right hepatic lobe and a fistula between the inflamed gallbladder and proximal duodenum (Images 1 and 2). Multiple, rim-calcified gallstones were visualized within the proximal jejunum (Image 3). No evidence of thromboembolic disease within the visceral arterial bed was identified. The patient was managed non-operatively with repeat CT imaging showing decreased inflammation over his one-week hospital stay.

## DISCUSSION

Cholecystoenteric fistula (CF) is defined as a spontaneous tract between an inflamed gallbladder and one or more parts of the adjacent gastrointestinal tract. Cholecystoduodenal fistula accounts for approximately 75–80% of all such fistulas.^1^,^3^ It is an uncommon complication of cholelithiasis with reported incidences ranging from 0.5–0.9%.^2^ Chronic cholecystitis is the primary etiology for as many as 75% of CF patients.^3^ Preoperative diagnosis is challenging due to the non-specific symptoms of CF when compared with cholecystitis. Initial management should focus on symptomatic treatment, antibiotics for concurrent cholecystitis, and surgical consultation. Gallstone ileus is a potential serious complication requiring surgical evaluation.^4^

**CPC-EM Capsule**

What do we already know about this clinical entity?

Cholecystoduodenal fistula (CDF) is a rare complication of gallbladder disease with chronic cholecystitis as the primary etiology.

What is the major impact of the image(s)?

These images clearly characterize CDF and will help raise awareness for this particular disease.

How might this improve emergency medicine practice?

This presentation emphasizes the need to maintain a broad differential diagnosis for abdominal pain out of proportion to exam, with CDF as a possible cause.

## References

[1] Glenn F, Reed C, Grafe WR (1981). Biliary enteric fistula. Surg Gynecol Obstet.

[2] Chowbey PK, Bandyopadhyay SK, Sharma A (2006). Laparoscopic management of cholecystoenteric fistulas. J Laparoendosc Adv Surg Tech A.

[3] Sharma A, Sullivan M, English H (1994). Laparoscopic repair of cholecystoduodenal fistulae. Surg Laparosc Endosc.

[4] Abou-Saif A, Al-Kawas FH (2002). Complications of gallstone disease: Mirizzi syndrome, cholecystocholedochal fistula, and gallstone ileus. Am J Gastroenterol.

